# Synovial Fluid Aspiration Should Not Be Routinely Performed during the Two-Stage Exchange of the Knee

**DOI:** 10.1155/2018/6720712

**Published:** 2018-06-12

**Authors:** Sebastian P. Boelch, Magnus Roth, Joerg Arnholdt, Maximilian Rudert, Martin Luedemann

**Affiliations:** Julius-Maximilians University Wuerzburg, Department of Orthopaedic Surgery, Koenig-Ludwig-Haus, Germany

## Abstract

**Purpose:**

Detection of infection persistence during the two-stage exchange of the knee for periprosthetic joint infection is challenging. Synovial fluid culture (SFC) and synovial white blood cell count (SWBCC) before joint reimplantation are widespread diagnostic means for this indication. The sensitivity and specificity of SFC and of SWBCC for infection persistence before planned reimplantation were evaluated.

**Methods:**

94 two-stage exchanges of the knee with synovial fluid aspiration performed after a drug holiday of at least 14 days and before reimplantation or spacer exchange (planned reimplantation) were retrospectively analyzed. Only cases with at least 3 intraoperative samples at planned reimplantation were included. SFC and SWBCC were compared to pathogen detection (SFC_(culture)_/SWBCC_(culture)_) and to histopathological signs of infection persistence (SFC_(histo)_/SWBCC_(histo)_) from intraoperative samples at planned reimplantation. For SFC, the sensitivity and specificity were calculated. For SWBCC, the optimal cut-off value with its sensitivity and specificity was calculated with the Youden-Index.

**Results:**

Sensitivity and specificity of SFC_(culture)_ were 0.0% and 98.9%. Sensitivity and specificity of SFC_(histo)_ were 3.4% and 100%. The optimal cut-off value for SWBCC_(culture)_ was 4450 cells/*μ*l with a sensitivity of 50.0% and a specificity of 86.5%. The optimal cut-off value for SWBCC_(histo)_ was 3250 cells/*μ*l with a sensitivity of 35.7% and a specificity of 92.9%.

**Conclusion:**

The detection of infection persistence remains challenging and a consented approach is lacking. The results do not warrant the routine performance of SFC during the two-stage exchange at the knee. SWBCC can be used to confirm infection persistence at high cut-offs, but they only occur in few patients and are therefore inappropriate for the routine use.

## 1. Introduction

Periprosthetic joint infection (PJI) is a devastating complication after total knee arthroplasty (TKA). Although the risk of PJI after primary TKA is reported as low as 0.5% to 1.9% [1], between 14.8% and 25.0% of TKA revisions are performed because of PJI [2–5]. With the expected increase of primary TKA, the absolute number of PJI will increase, too [6]. The two-stage exchange (TSE) is the most preferred treatment for PJI of the knee [1, 7]. The first stage of the TSE consists of prosthesis removal along with debridement of all infected tissue and commonly implantation of an antibiotic-loaded spacer. The first stage is followed by systemic antibiotic administration. At the second stage, a prosthesis is reimplanted or, in cases of infection persistence, the joint is redebrided, the spacer exchanged, and another course of systemic antibiotics administered. Infection persistence is assessed by means of clinical examination and blood infection markers such as the C-reactive protein (CRP). Synovial fluid culture (SFC) and synovial white blood cell count (SWBCC) gained from the affected joint by aspiration before the planned reimplantation (interstage aspiration (IA)) ought to help discriminating infection persistence from infection eradication. This evaluation of infection eradication with cultures from the joint before planned reimplantation is a well-established treatment algorithm [8]. Since the continuous administration of antibiotics until aspiration has been shown to reduce culture sensitivity of the SFC [9], an antibiotic free interval before IA, the so-called drug holiday, is recommended [1]. However, because of the drug holiday and the time until final results of the SFC are available, IA extends the duration until the second stage can be performed. A shorter interval to planned reimplantation may decrease soft tissue contraction, shorten immobilisation, and ultimately improve quality of life [10]. Thus, the routine implementation of the IA into the TSE has regained controversy [11], especially since recent studies showed a questionable clinical value.

Hoell et al. reported a sensitivity of the SFC by IA at hips and knees of only 5.0% [12]. Recent studies from hips with a girdlestone situation or with an indwelling spacer have confirmed this poor result with sensitivities between 4.3% and 30.0% [13, 14]. The study by Lonner et al. reported a sensitivity of 0.0% in 2001 from 34 TSEs of the knee [15].

Next to the SFC, the SWBCC may help to rule out infection persistence. However, derived from studies of the hip and studies of knees and hips, the optimal cut-off during the TSE is unclear and ranges between 640 and 2000 cells/*μ*l [12, 14, 16].

This study investigates the sensitivity of the SFC for infection persistence during the TSE at the knee under microbiological and histopathological considerations. Additionally, this is the first study to analyze the cut-off values of the SWBCC particularly at the knee.

## 2. Methods

### 2.1. Patient Inclusion

**Figure 1:**
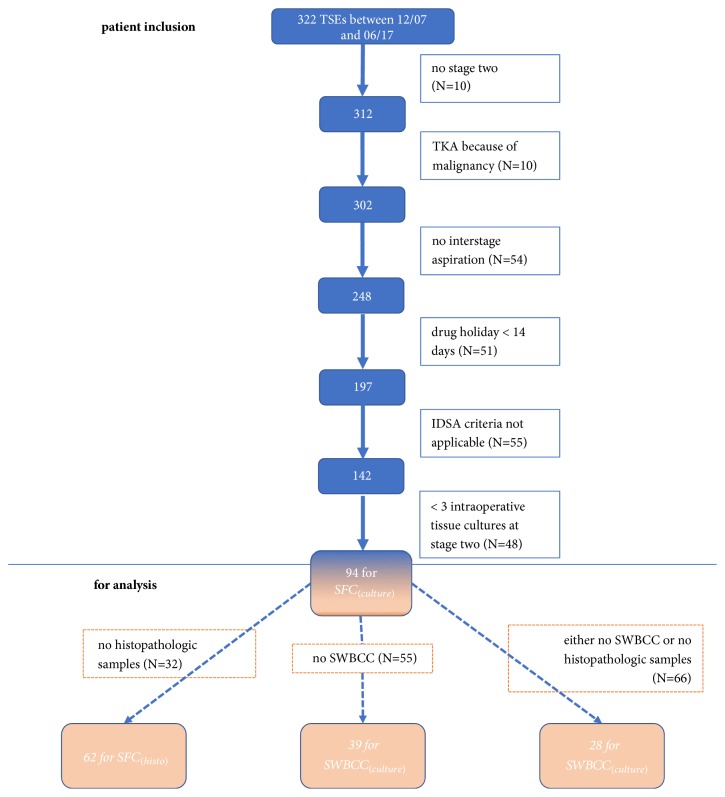
Flow chart of patient inclusion and numbers for analysis. TSE: two-stage exchange; TKA: total knee arthroplasty; IDSA: Infectious Disease Society of America; SFC: synovial fluid culture; SWBCC: synovial white blood cell count.

After approval by the institution's ethics review board, the electronic database of our orthopaedic department was retrospectively searched for all TSEs of the knee done between 12/07 and 06/17 (N=322). From these, 5 patients died before the second stage and further 5 denied reimplantation. 10 patients were excluded because primary TKA was done after tumor resection. 54 TSEs were excluded because IA was not performed. 51 TSEs were excluded because the drug holiday was less than 14 days. PJI was retrospectively defined according to the Clinical Practice Guidelines by the Infectious Disease Society of America as (a) sinus tract that communicates with the prosthesis; (b) presence of acute inflammation as seen on histopathologic examination of periprosthetic tissue at the time of prosthesis removal; (c) presence of purulence around the prosthesis; (d) two or more intraoperative cultures or combination of preoperative aspiration and intraoperative cultures that yield the same organism/or the growth of Staphylococcus aureus in a single specimen of synovial fluid or a tissue biopsy [7]. For this PJI definition further 55 cases had to be excluded. Finally, 48 TSEs were excluded because less than 3 intraoperative samples for microbiological evaluation were collected at the second stage, leaving 94 TSEs for analysis (Figure 1).

### 2.2. Treatment Regimen

Stage one of the TSE consisted of the removal of the prosthesis and debridement of infectious altered tissue and bone. In dependence of the soft tissue and bony situation an articulating or a static antibiotic-loaded polymethylmethacrylate spacer was implanted. The spacer was hand molded around Steinman spins as an endoskeleton from Palacos® R+G (Fa. Heraeus, Germany), a gentamicin premixed bone cement. 2 grams of vancomycin was additionally added per 40 cc batch of the bone cement. Antibiotics were administered for 4 to 8 weeks in dependence of pathogen detection and as recommended by the infectious specialist. Before this study, IA was performed by default after a drug holiday of 2 weeks under sterile conditions in an operating theater. After samples for the SFC were obtained, the remaining synovia was used for the SWBCC. If the SFC yielded no growth after 16 days of cultivation and the course of the CRP as well as clinical examination showed no persisting infection reimplantation was performed. Otherwise the spacer was exchanged. After reimplantation antibiotic treatment was continued for two weeks if tissue samples remained sterile. In case of pathogen detection antibiotic treatment was extended for 6 weeks as recommended by the infectious specialist.

### 2.3. Definitions and Statistics

Infection persistence was defined by microbiological and histopathological findings at planned reimplantation.

For infection persistence under a microbiological aspect, the SFC at IA was considered true positive, if it yielded the same pathogen detected from the intraoperative tissue samples at planned reimplantation (SFC_(culture)_). The SFC_(culture)_ was considered true negative, if it remained sterile and the intraoperative samples from planned reimplantation yielded less than two identical pathogens. The SFC_(culture)_ was considered false negative, if it remained sterile, but a virulent microorganism such as Staphylococcus aureus or Pseudomonas aeruginosa grew from at least one intraoperative tissue sample or a nonvirulent microorganism from at least two. These definitions are in accordance with the current recommendations by the Infectious Disease Society of America and Musculoskeletal Infectious Society [7, 17]. The SWBCC was considered true positive, if it was above the cut-off level and at least two intraoperative tissue samples yielded the same pathogen or one sample a virulent pathogen (SWBCC_(culture)_).

For infection persistence under a histopathological aspect, the SFC at IA was considered true positive, if it yielded a pathogen and intraoperative tissue samples at planned reimplantation showed histopathologic signs of infection (SFC_(histo)_). The SWBCC was considered true positive, if it was above the cut-off level and intraoperative tissue samples at planned reimplantation showed histopathologic infection persistence (SWBCC_(histo)_). Tissue samples showing acute inflammation as recommended by the Infectious Disease Society of America guideline [7] or periprosthetic membranes classified as type II or III according to Krenn and Morawietz [18] were regarded as infection persistence.

Sensitivity was defined as the number of true positive specimens/(true positive + false negative specimens), specificity as the number of true negative specimens/(true negative + false positive specimens), positive predictive value as the number of true positive specimens/(true positive + false positive specimens), and negative predictive value as the number of true negative specimens/(true negative + false negative specimens). Means were compared with the Mann–Whitney U test. P <0.05 was set statistically significant.

The optimal cut-off value for the SWBCCs was calculated with the Youden-Index after performing a receiver operating characteristics curve. All statistics were conducted with SPSS version 23 (SPSS Inc. Chicago, IL, USA).

## 3. Results

### 3.1. Patients and Treatment

 94 TSEs were included. The mean duration from index surgery to prosthesis removal and spacer implantation was 46.8 months (1 – 192) with 61.7% of the TSEs being repeated revisions. 47.9% were male patients. Mean age at TSE was 69.0 years (46 – 91) and the mean BMI was 31.0 kg/cm^2^ (20.5 – 48.1). Pathogens identified at PJI diagnosis are depicted in Table 1.

An articulating spacer was implanted in 75.5 % and a static spacer in 24.5%. The mean duration of intravenous antibiotic administration was 16.3 days (8 – 38) followed by a course of oral antibiotic therapy for a mean of 16.9 days (3 – 31). The mean drug holiday was 18.0 days (14 – 48). The mean duration from prosthesis removal to planned reimplantation was 74.0 days (54 – 147). Mean CRP before stage two was 1.1 mg/dl (0.00 – 10.00). In 6 patients, a spacer exchange was performed as second stage, in two because of intraoperative aspect of purulence, in one because of detection of Staphylococcus hominis at IA, and in 3 because of suspicious course of the CRP. 3.1 (3 – 4) microbiologic samples were taken at planned reimplantation.

### 3.2. Synovial Fluid Cultures from IA Compared to Microbiological Samples at Planned Reimplantation (*SFC*_(culture)_)

88 SFCs were true negative with sterile results from IA and the second stage. Further 3 sterile SFCs were considered true negative, although Staphylococcus epidermidis was cultivated from one single intraoperative sample. Microbiological infection persistence at reimplantation occurred in two cases: Staphylococcus epidermidis was cultured in 2 of 4 samples in the first and Staphylococcus epidermidis together with Pseudomonas aeruginosa from one of three samples in the second case. In both patients, the SFC was false negative. One SFC was false positive with growth of Staphylococcus hominis, but sterile samples at spacer exchange. The sensitivity of the SFC_(culture)_ was 0.0% (Table 2).

### 3.3. Synovial Fluid Cultures from IA Compared to Histopathologic Samples at Planned Reimplantation (*SFC*_(histo)_)

Histopathologic samples from the planned reimplantation were available in 62 TSEs. In 29 cases, tissue samples showed infection persistence (positive). In one of these cases, the SFC yielded a pathogen, which was Staphylococcus hominins (true positive). In the remaining 33 cases tissue samples showed no infection persistence and the SFCs were negative (true negative).

### 3.4. Synovial White Blood Cell Count at IA Compared to Microbiological Samples at Planned Reimplantation (*SWBCC*_(culture)_)

SWBCC results from 39 TSEs were available. The two cases with microbiological infection persistence had SWBCCs of 4800/*μ*l and of 600/*μ*l (positive). In one patient with a SWBCC of 500/*μ*l at IA one of three tissue cultures at planned reimplantation yielded Staphylococcus epidermidis (negative). The remaining 36 cases with a mean SWBCC of 2304/*μ*l (50 – 14000) had sterile tissue cultures at planned reimplantation (negative). The threshold with the highest Youden-Index (0.365) was 4450/*μ*l with a sensitivity of 50% and a specificity of 86.5%.

### 3.5. Synovial White Blood Cell Count from IA Compared to Histopathologic Samples at Planned Reimplantation (*SWBCC*_(histo)_)

**Figure 2:**
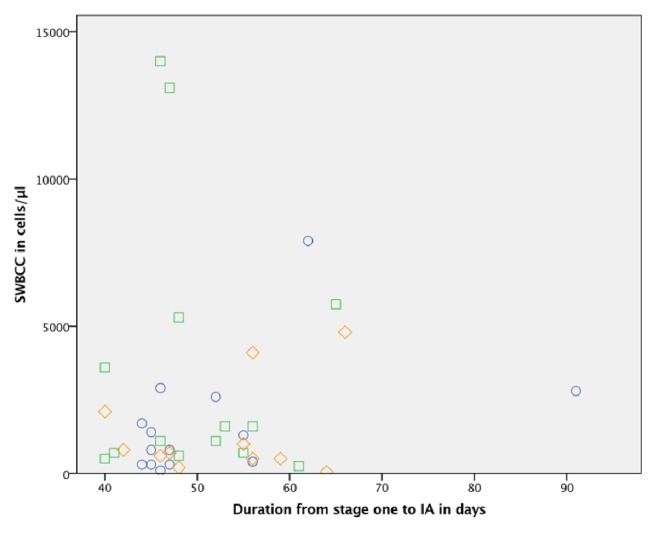
Correlation between synovial white blood cell count and duration from stage one to interstage aspiration. SWBCC: synovial white blood cell count; IA: interstage aspiration; circles showing SWBCC without histopathologic samples indicating infection persistence; squares showing SWBCC with histopathologic samples indicating infection persistence; diamonds showing SWBCC without histopathologic samples.

There was no correlation between the SWBCC and the duration from stage one to IA as shown in Figure 2. SWBCCs with the corresponding histopathologic samples from planned reimplantation were available in 28 cases.

In the 14 cases with histopathologic infection persistence the mean SWBCC was 3564/*μ*l (250 – 14000). Of these, one case yielded Staphylococcus epidermidis in one of three tissue cultures at reimplantation. The SWBCC was 600/*μ*l. In the remaining 14 cases without histopathologic infection persistence the mean SWBCC was 1686/*μ*l (100 – 7900). None of these cases had pathogen detection at planned reimplantation. We found no significant difference between SWBCCs with infection persistence or with infection eradication (p=0.329).

The SWBCC cut-off with the highest Youden-Index was 3250/*μ*l with a sensitivity of 35.7% and a specificity of 92.9% as shown in Table 3.

## 4. Discussion

We found insufficient sensitivity of the SFC for the routine performance during TSE in order to detect infection persistence. The high threshold of SWBCC_(culture)_ 4450/*μ*l had specificity of 86.5% but with a sensitivity of only 50%.

In spite of the retrospective design, the strengths of this study are the high number of cases, the strict adherence to the drug holiday, the sampling of at least three tissue specimens for culture at planned reimplantation as recommended by the Infectious Disease Society of America and Musculoskeletal Infectious Society, and the clear definition of infection persistence. However, several limitations need to be discussed.

So far, there is no uniform definition of infection persistence during the TSE. In the current study, infection persistence was defined under two different aspects: pathogen detection and histopathologic evaluation. As a clear limitation to this study, other features, that might indicate infection persistence such as pus and the CRP, were not considered. Newman et al. determined modified Musculoskeletal Infectious Society criteria for the definition of infection persistence at the hip [14]. Although only a small proportion of cases was diagnosed on the bases of minor criteria, it should be mentioned that the values of purulence or of the CRP as indicators for infection persistence have not been ultimately determined and remain controversial [11, 12, 19]. Additionally, more complex infection persistence definitions bear the risk of lacking traceability [13, 15]. In accordance with other authors, we defined infection persistence as detection of the same pathogen in at least two intraoperative cultures or detection of a virulent pathogen in a single [12, 16, 20]. But still, derived from the results of PJI diagnosis, infection can occur without pathogen detection in up to 24% [21]. As an alternate tool for PJI diagnosis [10, 22], histopathological evaluation was also investigated. In accordance with SFC_(culture)_ the sensitivities of SFC_(histo)_ and SWBCC_(histo)_ were very low, too. However, we noted a low consistency of cultures and histopathologic results at planned reimplantation. Only 6.9% of the cases with histopathologic infection persistence yielded a pathogen. Both these cases were Staphylococcus epidermidis in one of three intraoperative tissue cultures. Accordingly, the only culture positive IA was considered false positive for SFC_(culture)_ and true positive for SFC_(histo)_. Additionally, 91.7% of the culture positive PJIs at stage one were pathogen eradicated at stage two, but from histopathology negative PJI at initial diagnosis 68.4% were assessed with persistent infection at planned reimplantation. Histopathological studies indicate that neutrophil counts are substantially higher in case of infection persistence at stage two compared to initial PJI diagnosis [23]. This issue was recently highlighted by George et al. who demonstrated low sensitivity of frozen section for ruling out septic failure after reimplantation [24]. Thus, the criteria for histopathological analysis at planned reimplantation for evaluation of infection persistence clearly need clarification and validation.

Follow-up studies could confirm the low sensitivity of IA on the bases of reinfection, instead of clinical, microbiological, and histopathological findings at planned reimplantation. Zmistowski et al. defined infection persistence amongst other features by the need for septic revision due to same causative organism and reported a sensitivity of only 44% for the SWBCC [16]. However, under consideration of the statistic frequencies of causative organisms this approach is also limited by the inability to discriminate infection recurrence to new infection. It is agreed that pathogen detection and histopathologic evaluation are major columns with high sensitivities and specificities for PJI diagnosis [7, 17, 25, 26]. With our approach we must conclude that the value of IA for the question what to expect from sampling at planned reimplantation is insignificant. This conclusion is emphasized by the low sensitivities that have been recently reported for the hip or combined for the hip and the knee, irrespectively of the definition of infection persistence (Table 4) [12, 14, 20, 27]. The current study confirms these low sensitivities particularly at the knee.

Although we had comparable high numbers for microbiologic evaluation, a further limitation to this study is the few cases of infection persistence. But still, this study demonstrates that the SCF_(culture)_ failed to identify these patients. Owed to the fact that before this study we preferred performing the SFC over the SWBCC, the numbers for evaluating the optimal cut-off for the SWBCC are low. Thus, only if enough synovia was aspirated, SWBCC could be analyzed. For the SWBCC_(culture)_, the calculations are based on only two cases with culture positive infection persistence. Thus, our highest Youden-Index is rather low, and compared to the data derived from either hips or hips and knees, our calculated thresholds for SWBCC are high. The variation of thresholds and sensitivities may be attributed to statistical methods, different rates of infection persistence, and infection persistence definitions (Table 4). Although the thresholds for synovial leukocyte count proposed for PJI diagnosis by the Musculoskeletal Infection Society and by the European Bone and Joint Infections Society are well established, they should not replace the lack of a consented cut-off value at IA [11, 17, 28]. In case that SWBCC is preformed, the result needs to be interpreted with respect to clinical parameters and the patients' overall health condition. Low thresholds lead to low specificities and thus bear the risk of overtreatment with inadequate spacer exchange and another course of antibiotic administration. High levels can confirm infection persistence but are rare.

While the SFC adds no information to the question of infection persistence during the TSE, the SWBCC may help to confirm infection persistence in selected cases.

## 5. Conclusion

The detection of infection persistence remains challenging and a consented approach is lacking. The results do not warrant the routine performance of SFC during the two-stage exchange at the knee. SWBCC can be used to confirm infection persistence at high cut-offs, but they only occur in few patients and are therefore inappropriate for the routine use.

## Figures and Tables

**Table 1:** Pathogens detected at diagnosis of periprosthetic infection.

Pathogen	N (%)
Staphylococcus epidermidis	19 (20.21)
Staphylococcus aureus	15 (16.00)
Other CNS	13 (13.83)
Streptococci	5 (5.32)
Corynebacterium spp.	1 (1.1)
Escherichia coli	1 (1.1)
Enterobacter cloacae	1 (1.1)
Enterococcus faecalis	1 (1.1)
Bacillus cereus	1 (1.1)
Micrococcus luteus	1 (1.1)
Moraxella osloensis	1 (1.1)
MRSA	1 (1.1)
Cutibacterium acnes	1 (1.1)
Pseudomonas aeruginosa	1 (1.1)
Rothia dentocariosa	1 (1.1)
Polymicrobial	8 (8.51)
Culture-negative	23 (24.47)

CNS: coagulase negative staphylococci; MRSA: multi-resistant Staphylococcus aureus.

**Table 2:** Sensitivity and specificity of synovial fluid culture and synovial leukocyte count at interstage aspiration.

	Threshold	Sensitivity	Specificity	Positive Predictive Value	Negative Predictive Value
SFC_(culture)_	culture positive	0.000	0.989	0.000	0.978
SFC_(histo)_	culture positive	0.034	1.000	1.000	0.541
SWBCC_(culture)_	4450 cells/*μ*l	0.50	0.865	0.167	0.970
SWBCC_(histo)_	3250 cells/*μ*l	0.357	0.929	0.833	0.591

SFC: synovial fluid culture; SWBCC: synovial white blood cell count.

**Table 3:** Youden-Index in relation to synovial cell count with sensitivity and specificity for histopathologic infection persistence.

Youden-Index	SWBCC (cells/*μ*l)	Sensitivity	Specificity
0.000	99	1.000	0.000
0.071	175	1.000	0.071
0.000	275	0.929	0.071
0.214	350	0.929	0.286
0.286	450	0.929	0.357
0.214	550	0.857	0.357
0.143	650	0.786	0.357
0.000	750	0.643	0.357
0.143	950	0.643	0.500
0.000	1200	0.500	0.500
0.071	1350	0.500	0.571
0.143	1500	0.500	0.643
0.000	1650	0.357	0.643
0.071	2150	0.357	0.714
0.143	2700	0.357	0.786
0.214	2850	0.357	0.857
***0.286***	***3250***	***0.357***	***0.929***
0.214	4450	0.286	0.929
0.143	5525	0.214	0.929
0.071	6825	0.143	0.929
0.143	10500	0.143	1.000
0.071	13550	0.071	1.000
0.000	14000	0.000	1.000

SWBCC: synovial white blood cell count.

**Table 4:** Comparison of results for sensitivity and specificity for interstage aspiration by different authors.

Author	Definition of persistent infection	Joints	SFC		SWBCC		
			Sensitivity	Specificity	Cut-off	Sensitivity	Specificity
Hoell et al. 2016	at least two identical tissue cultures at SES	56 Hip- and 59 Knee-Spacers	0.05	0.99	970	0.313	0.391

Newman et al. 2017	modified MSIS at SES	77 Hip-Spacers	0.30	1.00	1166	0.76	0.78

Zmistowski et al. 2017	positive tissue culture at SES, and/or subsequent surgery for PJI after reimplantation	40 Hip- and 88 Knee-Spacers	-	-	1234	0.444	0.755

Muhlhofer et al. 2018	2 positive tissue cultures at SES	92 Hip- and Knee- Spacers (60 for SWBCC)	0.06	0.92	-	0.10	0.81

Boelch et al. 2018	modified IDSA criteria at SES	92 Hip-Spacers	0.05	0.94	2000	0.25	0.969

this study	histopathologic sign of infection persistence at SES	62 Knee-Spacers (28 for SWBCC)	0.03	1.00	3250	0.357	0.929
	at least two identical tissue cultures at SES or growth of a virulent microorganism	94 Knee-Spacers (39 for SWBCC)	0.00	0.99	4450	0.50	0.865

SFC: synovial fluid culture; SWBCC: synovial white blood cell count; SES: second stage; PJI: periprosthetic joint infection; MSIS: Musculoskeletal Infection Society.

## Data Availability

The datasets used during the current study are available from the corresponding author on reasonable request.
